# Positional relative age effect in europe’s top ten football leagues

**DOI:** 10.1186/s13102-025-01312-1

**Published:** 2025-08-30

**Authors:** Mücahit Işık, Mehmet Yavuz, Osman Dişçeken, Halil İbrahim Ceylan, Tugay Öksüz, Kadir Demir

**Affiliations:** 1 Department of Coaching Education, Faculty of Sport Sciences, Nevşehir Hacı Bektaş Veli University, Nevsehir, Orcid Turkey; 2 Physical Education and Sports Teaching Department, Faculty of Sports Sciences, Atatürk University, Erzurum, Turkey; 3 Master’s Student, Department of Training and Movement Sciences, Institute of Health Sciences, Gazi University, Ankara, Turkey; 4 Independent Researcher, Graduate of Coaching Education Department, Faculty of Sport Sciences, Nevşehir Hacı Bektaş Veli University, Nevşehir, 2024 Turkey

**Keywords:** Relative age effect (RAE), UEFA, Professional football, Playing position, Player selection, Top leagues, Elite sport

## Abstract

**Background:**

A previous study confirmed the existence of the relative age effect (RAE) in the top ten leagues of the Union of European Football Associations (UEFA) during the 2016–2017 season, reporting that the effect was particularly pronounced among defenders and midfielders. The present study investigates whether this effect persists. Specifically, it aims to examine the RAE among professional male footballers in UEFA’s top ten leagues during the 2023–2024 season, and to explore how the effect varies by playing position and league.

**Methods:**

The study analysed the birthdates and playing positions of 5,238 professional footballers. Birthdates were categorised into four quartiles: Q1 (January 1 to March 31), Q2 (April 1 to June 30), Q3 (July 1 to September 30), and Q4 (October 1 to December 31), and were examined in detail by league and position.

**Results:**

Players born in Q1 (30.3%) were overrepresented compared to those born in Q4 (20.5%) (χ² = 132.470, *p* < .05). This difference was statistically significant in most leagues, particularly in Serie A (χ² = 33.535, V = 0.14), Ligue 1 (χ² = 23.741, V = 0.11), LaLiga (χ² = 22.491, V = 0.12), and Bundesliga (χ² = 21.681, V = 0.12), but not in Premier League (χ² = 1.665, *p* = .645). In terms of playing position, the RAE was present across all groups (*p* < .05), with the most pronounced effect observed among goalkeepers (χ² = 40.006, V = 0.15). Positional RAE patterns varied across leagues: significant differences were found for goalkeepers in Serie A, Süper Lig, and LaLiga; for defenders in LaLiga and Jupiler Pro League; for midfielders in Serie A, Ligue 1, Süper Lig, and Eredivisie; and for forwards in Ligue 1, Bundesliga, and Scottish Premiership (*p* < .05).

**Conclusion:**

The findings indicate that the RAE persists in professional football within UEFA’s top leagues. The effect is evident across all playing positions and varies by league context.

## Background

Football is the most popular sport worldwide and is played in virtually every country [1]. In this sport, athletes’ physical [2, 3] and mental capacities [4], as well as their age [5], play a significant role in performance. Age may affect physical performance attributes, such as endurance, speed, and related capacities [5]. It may also affect their technical proficiency, depending on their positional role [6]. Previous studies have shown that the influence of age varies according to playing position [5, 7–10], league [9, 11], or level of competition [12–14]. Therefore, age is not only a determinant of overall performance but also a crucial factor in players’ ability to meet the physical and tactical demands of specific roles.

The physical and tactical demands of football differ across playing positions, such as goalkeeper, defenders, midfielders, and forwards [15]. Consequently, the optimal age for achieving peak performance may vary according to playing position [16]. For instance, goalkeepers and defenders often sustain high-level performance into older ages, whereas forwards typically reach their peak at a younger age [10, 17]. In terms of anthropometric characteristics, goalkeepers and central defenders typically possess greater height and body mass compared to players in other positions [18]. Such developmental and positional differences may offer an advantage in selection processes, particularly for those born earlier in the calendar year.

Performance differences can be observed even among players within the same age group [19–22], underscoring the necessity of examining the presence of the Relative Age Effect (RAE) [23–25]. RAE refers to the age gap between individuals born in the same calendar year but in different months [26]. This disparity may play a critical role in the career development of football players [27]. Supporting this perspective, previous research has demonstrated that players born early in the year enjoy advantages in selection and development processes, especially within youth categories [28, 29].

In European women’s football, early-born players are overrepresented, suggesting a structural imbalance related to birthdate [30]. Similar patterns are evident in elite youth football, where early-born players tend to have higher market values [31, 32]. However, this effect diminishes at the professional level, where attributes such as skill and performance become more decisive in determining market valuation [32–34].

Executive function performance in elite youth footballers has been found to correlate significantly with chronological age, while RAE and physical maturity showed no significant effect [35]. These findings suggest that chronological age should be prioritised when evaluating executive functions for talent identification, whereas RAE and physical maturity should be considered less decisive factors [35]. Although RAE may offer a selection advantage in highly competitive youth environments, this benefit seems to stem more from structural selection biases than from genuine physical superiority [36].

In a study conducted at an elite football academy in Spain, players born at the beginning of the year were overrepresented compared to those born later. However, birthdate did not significantly influence players’ chances of selection or promotion to elite levels, suggesting that RAE had no substantial effect on long-term developmental outcomes in that setting [37]. Among young Italian footballers, those born earlier in the year were more likely to be selected for youth national teams, whilst later-born players demonstrated a greater likelihood of ultimately reaching the senior national team. This pattern has been accounted for by two complementary theoretical perspectives: the Underdog Hypothesis and the knock-on effect. The Underdog Hypothesis posits that later-born players, having faced disadvantages during earlier selection stages, are forced to develop greater resilience and skill, which may contribute to long-term success. Conversely, the knock-on effect refers to residual selection bias, whereby early-born players appear more frequently at advanced levels simply because they were selected more often in earlier stages [38]. RAE appears to extend beyond youth categories, as players born in the first quarter of the year have been reported to reach elite levels more easily due to advantages accumulated during their development pathway [39].

Yamamoto et al. [40] conducted a study on professional footballers in the Japan Professional Football League (J-League) and concluded that the likelihood of becoming a footballer varies according to month of birth and birth order, suggesting that these factors may contribute to structural inequality. Early evidence of RAE in professional male football was identified in the German Bundesliga between the 1963/64 and 2006/07 seasons, where it was demonstrated to be a critical factor in attaining both player and coaching status [41]. RAE has also been investigated in relation to positional differences. In Turkish leagues, the distribution of players’ birth months differed by playing position, suggesting a positional influence of RAE [42]. Similarly, in major European professional leagues, RAE was found to be more pronounced among defenders and midfielders, although this varied across countries and competitions [43].

This study builds directly upon the foundational work of Yagüe et al. [44], who examined the presence of RAE in UEFA’s top ten leagues during the 2016–2017 season. While their research made a significant contribution to the understanding of RAE at the time, the current study aims to update and extend these findings by incorporating data from the 2023–2024 season, a period characterised by structural and contextual changes within European football. Not only has the composition of UEFA’s top ten leagues changed due to updated club coefficients, but this study also broadens the analytical scope by conducting detailed position-by-league comparisons. By doing so, it offers a more refined exploration of the RAE and its positional dynamics in contemporary professional football. Despite being a well-documented phenomenon, the continued investigation of the RAE remains essential to determine whether it persists under evolving structural and contextual conditions in modern football. This study explores how the RAE manifests across player positions and leagues, as well as within positions in each league.

By providing up-to-date insights into how relative age interacts with playing position and performance outcomes, this study aims to support the development of fairer and more effective strategies for talent identification and selection. In doing so, it seeks to contribute to the existing literature and promote a more balanced and inclusive structure within professional football.

## Methods

### Study design

This study employed a cross-sectional and descriptive design to investigate the presence of the RAE among professional male footballers competing in UEFA’s top ten leagues during the 2023–2024 season. The objective was to variations in RAE based on playing position (goalkeeper, defender, midfielder, forward) and league category.

### Participants

The sample comprised of 5,238 professional male footballers registered in the top ten UEFA-affiliated leagues: the Jupiler Pro League (Belgium), Eredivisie (Netherlands), Primeira Liga (Portugal), La Liga (Spain), Bundesliga (Germany), Serie A (Italy), Ligue 1 (France), Premier League (England), Süper Lig (Turkey), and Scottish Premiership (Scotland) (Table 1).

The distribution of players by playing position is provided in Table 2. All players were active during the 2023–2024 season and listed in official team rosters.

**Table 1:** Distribution of players according to country

Country	*N*	Percent (%)
Jupiler Pro League (Belgium)	534	10.2%
Eredivisie (Netherlands)	491	9.4%
Primeira Liga (Portugal)	495	9.5%
LaLiga (Spain)	507	9.7%
Bundesliga (Germany)	502	9.6%
Serie A (Italy)	550	10.5%
Ligue 1 (France)	607	11.6%
Premier League (England)	641	12.2%
Süper Lig (Turkey)	602	11.5%
Scottish Premiership (Scotland)	309	5.9%
**Total**	**5238**	**100.0%**

**Table 2:** Distribution of players according to playing positions

Game Position	*N*	Percent (%)
Goalkeeper	626	12.0%
Defender	1720	32.8%
Midfielder	1569	30.0%
Forward	1323	25.3%
**Total**	**5238**	**100.0%**

### Procedure

The top ten UEFA leagues were identified on 1 February 2024, based on the UEFA association club coefficients (https://www.uefa.com [45]). Player data were collected from the Transfermarkt (https://www.transfermarkt.com [46]) and verified using the official websites of each league. Final data collection was completed on 11 March 2024. Official league websites included: Premier League (https://www.premierleague.com [47]), La Liga (https://www.laliga.com [48]), Serie A (https://www.legaseriea.it [49]), Bundesliga (https://www.bundesliga.com [50]), Ligue 1 (https://ligue1.com [51]), Eredivisie (https://eredivisie.eu [52]), Liga Portugal (https://www.ligaportugal.pt [53]), Jupiler Pro League (https://www.proleague.be [54]), Süper Lig (https://www.tff.org [55]), and Scottish Premiership (https://spfl.co.uk [56]).

The players’ birthdates (day, month, year), playing positions (goalkeeper, defender, midfielder, forward), and league affiliations were recorded in an Excel database. Birthdates were grouped into four calendar-year quartiles: Q1 (1 January–31 March), Q2 (1 April–30 June), Q3 (1 July–30 September), and Q4 (1 October–31 December).

Analyses began with an overall comparison of the quartile birthdate distributions across all leagues, regardless of playing position (Table 3), followed by comparisons by playing position across all leagues combined (Table 4). Finally, quartile distributions were analysed by position within each league: goalkeepers (Table 5), defenders (Table 6), midfielders (Table 7), and forwards (Table 8).

### Statistical analysis

Statistical analyses were conducted using IBM SPSS Statistics 21.0 (IBM Corp., Armonk, NY, USA). Cross-tabulations and chi-square (χ²) tests were used to examine associations between birth quartiles, playing positions, and league affiliation. Degrees of freedom (df) were calculated as (number of rows – 1) × (number of columns – 1), with statistical significance set at *p* < .05.

 Moreover, odds ratios (ORs) and 95% confidence intervals (CIs) were calculated to estimate the relative likelihood of players born in Q1, Q2, and Q3 being represented in each league or position compared to Q4.

Additionally, the relationships between league affiliation, playing position, and birth quartile were evaluated using chi-square tests, Cramér’s V, and OR estimations. Cramér’s V was used to assess the strength of associations. Classification of effect sizes for categorical variables was based on Cramér’s V, with values of 0.06–0.17 indicating a small effect, 0.18–0.29 indicating a moderate effect, and values greater than 0.30 indicating a large effect [44].

## Results

In this study, the birth quarter distributions of footballers competing in the top UEFA leagues were analysed. Table 3 displays the distribution of players across the leagues by birth quarter, together with the associated levels of statistical significance. The calendar year was divided into four quarters: the first quarter (Q1), second quarter (Q2), third quarter (Q3), and fourth quarter (Q4).

**Table 3:** Quarterly distribution of birth dates and odds ratios of players across different leagues

League		Q1	Q2	Q3	Q4	Total	χ²	df	V	*p*	OR (95% CI)		
**Total**	N	1589	1427	1148	1074	5238	132.470	27	0.09	0.000*	**Q1- Q4**	**Q2- Q4**	**Q3- Q4**
	%	30.3%	27.2%	21.9%	20.5%	100.0%							
Jupiler Pro League (Belgium)	N	158	151	118	107	534	13.850	3	0.09	0.003*	1.477 (1.357–1.608)	1.411 (1.296–1.536)	1.103 (1.013–1.201)
	%	29.6%	28.3%	22.1%	20.0%	100.0%							
Eredivisie (Netherlands)	N	137	146	105	103	491	11.802	3	0.09	0.008*	1.330 (1.217–1.453)	1.417 (1.297–1.548)	1.019 (0.933–1.113)
	%	27.9%	29.7%	21.4%	21.0%	100.0%							
Primeira Liga (Portugal)	N	153	141	105	96	495	18.382	3	0.11	0.000*	1.594 (1.460–1.741)	1.469 (1.345–1.604)	1.094 (1.002–1.195)
	%	30.9%	28.5%	21.2%	19.4%	100.0%							
LaLiga (Spain)	N	167	134	108	98	507	22.491	3	0.12	0.000*	1.704 (1.562–1.859)	1.367 (1.253–1.491)	1.102 (1.010–1.202)
	%	32.9%	26.4%	21.3%	19.3%	100.0%							
Bundesliga (Germany)	N	151	144	123	84	502	21.681	3	0.12	0.000*	1.798 (1.647–1.962)	1.714 (1.570–1.871)	1.464 (1.341–1.598)
	%	30.1%	28.7%	24.5%	16.7%	100.0%							
Serie A (Italy)	N	192	138	118	102	550	33.535	3	0.14	0.000*	1.882 (1.731–2.046)	1.353 (1.245–1.471)	1.157 (1.064–1.258)
	%	34.9%	25.1%	21.5%	18.5%	100.0%							
Ligue 1 (France)	N	192	169	127	119	607	23.741	3	0.11	0.000*	1.613 (1.490–1.747)	1.420 (1.311–1.538)	1.067 (0.985–1.155)
	%	31.6%	27.8%	20.9%	19.6%	100.0%							
Premier League (England)	N	171	157	149	164	641	1.665	3	0.03	0.645	1.043 (0.965–1.127)	0.957 (0.836–1.034)	0.909 (0.841–0.982)
	%	26.7%	24.5%	23.2%	25.6%	100.0%							
Süper Lig (Turkey)	N	177	163	137	125	602	11.236	3	0.08	0.011*	1.416 (1.307–1.534)	1.304 (1.204–1.412)	1.096 (1.012–1.187)
	%	29.4%	27.1%	22.8%	20.8%	100.0%							
Scottish Premiership (Scotland)	N	91	84	58	76	309	7.854	3	0.09	0.049*	1.197 (1.071–1.338)	1.105 (0.988–1.235)	0.763 (0.682–0.853)
	%	29.4%	27.2%	18.8%	24.6%	100.0%							

*: marks significant differences (*p* < .05); χ²: chi square; df: degrees of freedom; p: level of significance; V: Cramer´s V; OR: Odds Ratio; CI: Confidence Interval; %: percentage

When the birth dates of 5,238 players were analysed by quartile, the highest proportion was observed in Q1 (30.3%), while the lowest was in Q4 (20.5%) (Table 3). The results indicate that the overall distribution across birth quarters is statistically significant (χ² = 132.470, *p* < .05). This finding was further supported by the odds ratio and a small Cramer’s V (0.09), indicating a weak association. According to this value, the association between birth quarter and player representation in the leagues is weak. When each league was analysed individually (Table 3), the proportion of players born in Q1 was significantly higher than those born in Q4 in most leagues (*p* < .05). This difference was particularly marked in the LaLiga, Bundesliga, Serie A, and Ligue 1 . Conversely, no significant difference was found in the Premier League (χ² = 1.665, *p* = .645). According to odds ratio analyses, in the leagues where there were significant differences existed, players born in Q1 were more likely to be represented than those born in Q4. In the Bundesliga, players born in Q2 were more likely to be represented than those born in Q4 (OR = 1.417, 95% CI: 1.297–1.548). The strongest effect was observed for the Serie A (V = 0.14).

**Table 4:** Quarterly distribution of birth dates by playing position and associated odds ratios in football

Positions		Q1	Q2	Q3	Q4	Total	χ²	df	V	*p*	OR (95% CI)		
**Total**	N	1589	1427	1148	1074	5238	132.470	9	0.09	0.000*	**Q1-Q4**	**Q2- Q4**	**Q3- Q4**
	%	30.3%	27.2%	21.9%	20.5%	100.0%							
Goalkeeper	N	206	183	129	108	626	40.006	3	0.15	0.000*	1.907 (1.763–2.062)	1.694 (1.566–1.832)	1.194 (1.104–1.291)
	%	32.9%	29.2%	20.6%	17.3%	100.0%							
Defender	N	503	478	368	371	1720	34.786	3	0.08	0.000*	1.356 (1.293–1.422)	1.288 (1.229–1.350)	0.992 (0.946–1.040)
	%	29.2%	27.8%	21.4%	21.6%	100.0%							
Midfielder	N	486	408	347	328	1569	38.783	3	0.09	0.000*	1.482 (1.410–1.557)	1.244 (1.184–1.307)	1.058 (1.007–1.112)
	%	31.0%	26.0%	22.1%	20.9%	100.0%							
Forward	N	394	358	304	267	1323	28.791	3	0.09	0.000*	1.476 (1.399–1.558)	1.341 (1.271–1.415)	1.139 (1.079–1.202)
	%	29.8%	27.1%	23.0%	20.2%	100.0%							

*: marks significant differences (*p* < .05); χ²: chi square; df: degrees of freedom; p: level of significance; V: Cramer´s V; OR: Odds Ratio; CI: Confidence Interval; %: percentage

When the birth quartiles of the players were compared by playing positions (Table 4), statistically significant differences were observed in the distribution of birth dates across all positions (*p* < .05). Overall, players born in Q1 were more frequently represented than those born in Q4. This disparity was most pronounced among goalkeepers (χ² = 40.006, V = 0.15), with 32.9% of goalkeepers born in Q1 compared to just 17.3% in Q4 (OR = 1.907, 95% CI: 1.763–2.062). A similar, albeit less pronounced, trend was statistically evident among defenders (χ² = 34.786), midfielders (χ² = 38.783), and forwards (χ² = 28.791). Cramer’s V values indicated a small to moderate effect size across all positions.

**Table 5:** Quarterly distribution of birth dates by goalkeeper position and associated odds ratios across leagues

Goalkeeper		Q1	Q2	Q3	Q4	Total	χ²	df	V	*p*	OR (95% CI)		
**Total**	N	206	183	129	108	626	40.006	27	0.15	0.000*	**Q1-Q4**	**Q2-Q4**	**Q3-Q4**
	%	32.9%	29.2%	20.6%	17.3%	100.0%							
Jupiler Pro League (Belgium)	N	25	19	18	11	73	5.411	3	0.16	0.144	2.273 (1.807–2.859)	1.727 (1.373–2.172)	1.636 (1.301–2.058)
	%	34.2%	26.0%	24.7%	15.1%	100.0%							
Eredivisie (Netherlands)	N	21	20	13	13	67	3.388	3	0.13	0.336	1.615 (1.271–2.052)	1.538 (1.210–1.954)	1.000 (0.787–1.271)
	%	31.3%	29.9%	19.4%	19.4%	100.0%							
Primeira Liga (Portugal)	N	21	15	11	10	57	5.246	3	0.18	0.155	2.100 (1.620–2.722)	1.500 (1.157–1.945)	1.100 (0.848–1.426)
	%	36.8%	26.3%	19.3%	17.5%	100.0%							
LaLiga (Spain)	N	18	15	8	6	47	8.234	3	0.24	0.041*	3.000 (2.254–3.993)	2.500 (1.878–3.327)	1.333 (1.002–1.774)
	%	38.3%	31.9%	17.0%	12.8%	100.0%							
Bundesliga (Germany)	N	14	22	16	12	64	3.500	3	0.14	0.321	1.167 (0.913–1.491)	1.833 (1.435–2.342)	1.333 (1.043–1.703)
	%	21.9%	34.4%	25.0%	18.8%	100.0%							
Serie A (Italy)	N	28	17	17	7	69	12.797	3	0.25	0.005*	4.000 (3.159–5.064)	2.429 (3.498–5.608)	2.429 (1.918–3.075)
	%	40.6%	24.6%	24.6%	10.1%	100.0%							
Ligue 1 (France)	N	21	23	14	12	70	4.857	3	0.15	0.183	1.750 (1.385–2.212)	1.917 (1.517–2.423)	1.167 (0.923–1.475)
	%	30.0%	32.9%	20.0%	17.1%	100.0%							
Premier League (England)	N	23	19	14	22	78	2.513	3	0.10	0.473	1.045 (0.861–1.269)	0.864 (0.712–1.049)	0.636 (0.524–0.772)
	%	29.5%	24.4%	17.9%	28.2%	100.0%							
Süper Lig (Turkey)	N	24	23	13	9	69	9.551	3	0.21	0.023*	2.667 (2.071–3.435)	2.556 (1.985–3.292)	1.444 (1.121–1.860)
	%	34.8%	33.3%	18.8%	13.0%	100.0%							
Scottish Premiership (Scotland)	N	11	10	5	6	32	3.250	3	0.18	0.355	1.833 (1.296–2.592)	1.667 (1.179–2.357)	0.833 (0.589–1.178)
	%	34.4%	31.3%	15.6%	18.8%	100.0%							

*: marks significant differences (*p* < .05); χ²: chi square; df: degrees of freedom; p: level of significance; V: Cramer´s V; OR: Odds Ratio; CI: Confidence Interval; %: percentage

The quartile distributions of goalkeepers’ birth dates were compared (Table 5), and the overall analysis revealed a statistically significant difference (χ² = 40.006, *p* < .05). When goalkeepers were examined by league, significant differences in the birth quartile distributions were found in Serie A (χ² = 12.797), Süper Lig (χ² = 9.551), and LaLiga (χ² = 8.234) (*p* < .05). In these leagues, goalkeepers born in Q1 were notably more likely to be represented than those born in Q4. Among the leagues with significant differences, Cramer’s V values indicated a moderate association (V = 0.21–0.25), with the strongest effect observed in the Serie A (V = 0.25).

**Table 6:** Quarterly distribution of birth dates by defender position and associated odds ratios across leagues

Defender		Q1	Q2	Q3	Q4	Total	χ²	df	V	*p*	OR (95% CI)		
**Total**	N	503	478	368	371	1720	34.786	27	0.08	0.000*	**Q1- Q4**	**Q2- Q4**	**Q3- Q4**
	%	29.2%	27.8%	21.4%	21.6%	100.0%							
Jupiler Pro League (Belgium)	N	49	56	29	33	167	11.850	3	0.15	0.008*	1.485 (1.276–1.728)	1.697 (1.458–1.975)	0.879 (0.755–1.023)
	%	29.3%	33.5%	17.4%	19.8%	100.0%							
Eredivisie (Netherlands)	N	51	42	29	34	156	7.128	3	0.12	0.068	1.500 (1.282–1.755)	1.235 (1.056–1.445)	0.853 (0.729–0.998)
	%	32.7%	26.9%	18.6%	21.8%	100.0%							
Primeira Liga (Portugal)	N	52	50	34	32	168	7.810	3	0.12	0.050	1.625 (1.397–1.890)	1.563 (1.344–1.818)	1.063 (0.914–1.237)
	%	31.0%	29.8%	20.2%	19.0%	100.0%							
LaLiga (Spain)	N	64	50	32	28	174	19.195	3	0.19	0.000*	2.286 (1.970–2.652)	1.786 (1.539–2.072)	1.143 (0.985–1.326)
	%	36.8%	28.7%	18.4%	16.1%	100.0%							
Bundesliga (Germany)	N	55	46	40	30	171	7.737	3	0.12	0.052	1.833 (1.578–2.129)	1.533 (1.320–1.781)	1.333 (1.147–1.549)
	%	32.2%	26.9%	23.4%	17.5%	100.0%							
Serie A (Italy)	N	57	41	39	39	176	5.182	3	0.10	0.159	1.462 (1.261–1.695)	1.051 (0.907–1.218)	1.000 (0.863–1.159)
	%	32.4%	23.3%	22.2%	22.2%	100.0%							
Ligue 1 (France)	N	50	55	44	45	194	1.588	3	0.05	0.662	1.111 (0.965–1.279)	1.222 (1.062–1.407)	0.978 (0.850–1.126)
	%	25.8%	28.4%	22.7%	23.2%	100.0%							
Premier League (England)	N	56	53	54	55	218	0.092	3	0.01	0.993	1.018 (0.891–1.163)	0.964 (0.844–1.101)	0.982 (0.860–1.121)
	%	25.7%	24.3%	24.8%	25.2%	100.0%							
Süper Lig (Turkey)	N	45	60	47	46	198	3.010	3	0.07	0.390	0.978 (0.851–1.124)	1.304 (1.134–1.499)	1.022 (0.889–1.175)
	%	22.7%	30.3%	23.7%	23.2%	100.0%							
Scottish Premiership (Scotland)	N	24	25	20	29	98	1.673	3	0.08	0.643	0.828 (0.681–1.007)	0.862 (0.709–1.049)	0.690 (0.567–0.839)
	%	24.5%	25.5%	20.4%	29.6%	100.0%							

*: marks significant differences (*p* < .05); χ²: chi square; df: degrees of freedom; p: level of significance; V: Cramer´s V; OR: Odds Ratio; CI: Confidence Interval; %: percentage

The quartile distributions of defenders’ birth dates were compared (Table 6), and the overall analysis revealed a statistically significant difference (χ² = 34.786, *p* < .05). When examined by league, significant differences in the birth quartile distributions of defenders were observed in LaLiga (χ² = 19.195) and Jupiler Pro League (χ² = 11.850) (*p* < .05). In the LaLiga, players born in Q1 were more likely to be represented than those born in Q4 (OR = 2.286, 95% CI: 1.970–2.652), whereas in the Jupiler Pro League, players born in Q2 were more likely to be represented than those born in Q4 (OR = 1.697, 95% CI: 1.458–1.975). Cramer’s V values indicated a moderate association in LaLiga (V = 0.19), whereas the effect observed in the Jupiler Pro League (V = 0.15) was small. By contrast, the results for the Premier League showed no statistically significant difference in quartile distribution (χ² = 0.092, *p* = .993), and the corresponding odds ratios were very low, suggesting no meaningful disparity across birth quartiles.

**Table 7:** Quarterly distribution of birth dates by midfielder position and associated odds ratios across leagues

Midfielder		Q1	Q2	Q3	Q4	Total	χ²	df	V	*p*	OR (95% CI)		
**Total**	N	486	408	347	328	1569	38.783	27	0.09	0.000*	**Q1- Q4**	**Q2- Q4**	**Q3- Q4**
	%	31.0%	26.0%	22.1%	20.9%	100.0%							
Jupiler Pro League (Belgium)	N	49	45	48	35	177	2.774	3	0.07	0.428	1.400 (1.208–1.622)	1.286 (1.110–1.490)	1.371 (1.183–1.589)
	%	27.7%	25.4%	27.1%	19.8%	100.0%							
Eredivisie (Netherlands)	N	33	49	25	30	137	9.423	3	0.15	0.024*	1.100 (0.930–1.301)	1.633 (1.381–1.931)	0.833 (0.705–0.985)
	%	24.1%	35.8%	18.2%	21.9%	100.0%							
Primeira Liga (Portugal)	N	41	31	29	27	128	3.625	3	0.10	0.305	1.519 (1.277–1.806)	1.148 (0.965–1.365)	1.074 (0.903–1.277)
	%	32.0%	24.2%	22.7%	21.1%	100.0%							
LaLiga (Spain)	N	48	32	31	30	141	6.206	3	0.12	0.102	1.600 (1.357–1.887)	1.067 (0.905–1.258)	1.033 (0.876–1.218)
	%	34.0%	22.7%	22.0%	21.3%	100.0%							
Bundesliga (Germany)	N	39	41	30	26	136	4.529	3	0.11	0.210	1.500 (1.268–1.775)	1.577 (1.333–1.866)	1.154 (0.975–1.365)
	%	28.7%	30.1%	22.1%	19.1%	100.0%							
Serie A (Italy)	N	65	42	32	29	168	19.000	3	0.19	0.000*	2.241 (1.927–2.607)	1.448 (1.245–1.684)	1.103 (0.948–1.283)
	%	38.7%	25.0%	19.0%	17.3%	100.0%							
Ligue 1 (France)	N	66	54	38	37	195	11.827	3	0.14	0.008*	1.784 (1.550–2.053)	1.459 (1.268–1.679)	1.027 (0.893–1.182)
	%	33.8%	27.7%	19.5%	19.0%	100.0%							
Premier League (England)	N	53	51	52	54	210	0.095	3	0.01	0.992	0.981 (0.857–1.123)	0.944 (0.825–1.081)	0.963 (0.841–1.102)
	%	25.2%	24.3%	24.8%	25.7%	100.0%							
Süper Lig (Turkey)	N	58	39	40	29	166	10.530	3	0.15	0.015*	2.000 (1.704–2.347)	1.345 (1.146–1.578)	1.379 (1.175–1.618)
	%	34.9%	23.5%	24.1%	17.5%	100.0%							
Scottish Premiership (Scotland)	N	34	24	22	31	111	3.486	3	0.10	0.323	1.097 (0.911–1.321)	0.774 (0.643–0.932)	0.710 (0.589–0.855)
	%	30.6%	21.6%	19.8%	27.9%	100.0%							

*: marks significant differences (*p* < .05); χ²: chi square; df: degrees of freedom; p: level of significance; V: Cramer´s V; OR: Odds Ratio; CI: Confidence Interval; %: percentage

The quartile distributions of midfielders’ birth dates were compared (Table 7), and the overall analysis revealed a statistically significant difference (χ² = 38.783, *p* < .05). When analysed by league, significant differences in the birth quartile distribution of midfielders were found in Serie A (χ² = 19.000), Ligue 1 (χ² = 11.827), Süper Lig (χ² = 10.530), and Eredivisie (χ² = 9.423) (*p* < .05). In general, players born in Q1 were more likely to be represented in these leagues compared to those born in Q4. However, in the Bundesliga, players born in Q2 were more likely to be represented than those born in Q4 (OR = 1.633, 95% CI: 1.381–1.931). Among the leagues with significant differences, Cramer’s V values indicated predominantly small associations, with the only moderate effect observed in Serie A (V = 0.19).

**Table 8:** Quarterly distribution of birth dates by forward position and associated odds ratios across leagues

Forward		Q1	Q2	Q3	Q4	Total	χ²	df	V	*p*	OR (95% CI)		
**Total**	N	394	358	304	267	1323	28.791	27	0.09	0.000*	**Q1- Q4**	**Q2- Q4**	**Q3- Q4**
	%	29.8%	27.1%	23.0%	20.2%	100.0%							
Jupiler Pro League (Belgium)	N	35	31	23	28	117	2.624	3	0.09	0.453	1.250 (1.043–1.498)	1.107 (0.924–1.327)	0.821 (0.685–0.984)
	%	29.9%	26.5%	19.7%	23.9%	100.0%							
Eredivisie (Netherlands)	N	32	35	38	26	131	2.405	3	0.08	0.493	1.231 (1.037–1.461)	1.346 (1.134–1.597)	1.462 (1.232–1.735)
	%	24.4%	26.7%	29.0%	19.8%	100.0%							
Primeira Liga (Portugal)	N	39	45	31	27	142	5.493	3	0.11	0.139	1.444 (1.225–1.702)	1.667 (1.414–1.965)	1.148 (0.974–1.353)
	%	27.5%	31.7%	21.8%	19.0%	100.0%							
LaLiga (Spain)	N	37	37	37	34	145	0.186	3	0.02	0.980	1.088 (0.925–1.280)	1.088 (0.925–1.280)	1.088 (0.925–1.280)
	%	25.5%	25.5%	25.5%	23.4%	100.0%							
Bundesliga (Germany)	N	43	35	37	16	131	12.481	3	0.18	0.006*	2.688 (2.265–3.190)	2.188 (1.844–2.597)	2.313 (1.949–2.745)
	%	32.8%	26.7%	28.2%	12.2%	100.0%							
Serie A (Italy)	N	42	38	30	27	137	4.226	3	0.10	0.238	1.556 (1.316–1.840)	1.407 (1.190–1.663)	1.111 (0.940–1.314)
	%	30.7%	27.7%	21.9%	19.7%	100.0%							
Ligue 1 (France)	N	55	37	31	25	148	13.622	3	0.18	0.003*	2.200 (1.873–2.585)	1.480 (1.260–1.739)	1.240 (1.055–1.457)
	%	37.2%	25.0%	20.9%	16.9%	100.0%							
Premier League (England)	N	39	34	29	33	135	1.504	3	0.06	0.681	1.182 (1.007–1.388)	1.030 (0.877–1.209)	0.879 (0.749–1.032)
	%	28.9%	25.2%	21.5%	24.4%	100.0%							
Süper Lig (Turkey)	N	50	41	37	41	169	2.148	3	0.07	0.542	1.220 (1.049–1.419)	1.000 (0.860–1.163)	0.902 (0.776–1.049)
	%	29.6%	24.3%	21.9%	24.3%	100.0%							
Scottish Premiership (Scotland)	N	22	25	11	10	68	10.235	3	0.22	0.017*	2.200 (1.855–2.609)	2.500 (2.108–2.965)	1.100 (0.927–1.305)
	%	32.4%	36.8%	16.2%	14.7%	100.0%							

*: marks significant differences (*p* < .05); χ²: chi square; df: degrees of freedom; p: level of significance; V: Cramer´s V; OR: Odds Ratio; CI: Confidence Interval; %: percentage

The quartile distributions of forwards’ birth dates were compared (Table 8), and the overall analysis revealed a statistically significant difference (χ² = 28.791, *p* < .05). When analysed by league, significant differences in the distribution of the birth quarters among forwards were identified in Ligue 1 (χ² = 13.622), Bundesliga (χ² = 12.481), and Scottish Premiership (χ² = 10.235) (*p* < .05). In the Ligue 1 (OR = 2.200, 95% CI: 1.873–2.585) and Bundesliga (OR = 2.688, 95% CI: 2.265–3.190) leagues, forwards born in Q1 were more likely to be represented than those born in Q4. In contrast, in the Scottish Premiership, players born in Q2 were more likely to be represented than those born in Q4 (OR = 2.500, 95% CI: 2.108–2.965). Among the leagues with significant differences, Cramer’s V values indicate a moderate association (V = 0.18–0.22), with the strongest effect observed in the Scottish Premiership (V = 0.22).

## Discussion

This study examined the presence of the RAE among professional male footballers in UEFA’s top ten leagues during the 2023–2024 season. It also assessed how birthdate distributions varied by playing position (goalkeeper, defender, midfielder, forward) and across different leagues. The results showed that players born in the first quarter of the year (Q1; 30.3%) were overrepresented compared to those born in the fourth quarter (Q4; 20.5%). Although statistically significant, the overall effect size was small (Cramér’s V = 0.09), indicating a modest practical impact. These findings confirm the continued presence of the RAE in elite European football. The results align with the earlier work of Yagüe et al. [44], who identified similar patterns during the 2016–2017 season.

These findings are consistent with previous literature. Rodríguez-Lorenzo and Martín-Acero [57] found that, at Real Club Deportivo de la Coruña, the RAE influenced progression to the professional level, though not match participation. Similarly, Işıkdemir et al. [58] found higher representation of Q1-born players (32%) in both the UEFA Champions League and Europa League, compared to Q4-born players (19.4%). Mujika et al. [28] reported a consistent RAE across competitive levels at Athletic Club Bilbao, with the strongest effect among elite youth. Götze and Hoppe [59] identified the RAE among German male and female footballers, particularly in men’s second divisions and U-19 teams, while Peña-González et al. [36] showed a progressively stronger RAE across U-13 to U-15 levels in Spain’s youth divisions.

These patterns may be partially explained by the knock-on effect, a form of residual bias whereby early selection advantages during youth reinforce continued developmental opportunities into adulthood, thereby contributing to the persistence of the RAE at the professional level [38]. Hurley et al. [60] reported RAE variations across playing positions and nationalities in NCAA Division I football. In contrast, González-Víllora, Pastor-Vicedo, and Cordente [61] found no RAE at the professional level, though effects were significant in younger categories. Gürkan and Yıldırım [42] observed the RAE to be more pronounced in lower-tier Turkish leagues.

The underdog hypothesis offers a possible explanation for long-term success among relatively younger athletes. A longitudinal English academy study found that although early-born players were more likely to be selected, late-born players had greater odds of securing professional contracts [62]. Similarly, Castillo et al. [37] found that while early-born players were overrepresented in selection processes in a Spanish academy, birth date did not predict promotion to higher levels—emphasising the limitations of physical criteria in talent identification.

Although this study focused on male footballers, previous research has shown that RAE can vary by sex. Sedano, Vaeyens, and Redondo [63] found a stronger RAE among elite Spanish female players at higher competition levels. Similarly, Pérez-González et al. [30] observed the RAE in top woman’s leagues in Spain, Italy, England, and France, but not in Germany. In a global analysis of the 2023 FIFA Women’s World Cup, the RAE was only evident among European players [64]. Likewise, studies in handball [65], volleyball [66], and futsal [67] highlight that RAE expression varies by sex and sport.

In this study, the RAE was significant in all leagues except Premier League. An exception was also noted in the Eredivisie, where Q2-born players were more common than those born in Q4. Effect sizes across leagues were small, ranging from Cramér’s V = 0.08 to 0.14. Yagüe et al. [44], similarly found strong Q1 and Q2 representation, although in their study, Eerste Klasse A lacked statistical significance. These consistent trends point to the persistent nature of the RAE in elite football.

In the present study, no statistically significant RAE was found in the Premier League. One plausible reason for this absence could be the “birthday-banding” method. This strategy assigns players to age categories based on actual birthdays, avoiding rigid calendar-year boundaries. Kelly et al. [68] found no RAE across athlete development stages in squash using this method, suggesting its potential to neutralise RAE effects.

The literature also supports the RAE’s presence in youth football across Europe [24, 69, 70] and beyond [71]. Even when Q1 players are more likely to be selected into academies, Q4 players often achieve longer-term success—again reinforcing the underdog hypothesis [62].

The findings of the present study demonstrate that the RAE varies by playing position. Across all positions, players born in Q1 were overrepresented compared to those born in Q4, with the most pronounced disparity observed among goalkeepers. The RAE effect size was largest among goalkeepers (Cramér’s V = 0.15, approaching moderate), while other positions showed small effect sizes. These results align closely with those reported by Yagüe et al. [44], who identified the presence of the RAE across all positions, with the strongest associations found among midfielders, defenders, goalkeepers, and forwards. In the current study, the highest effect sizes were recorded in goalkeepers, followed by midfielders, defenders, and forwards, respectively.

In contrast to Yagüe et al. [44], who identified positional RAE effects without providing league-specific breakdowns, the present study offers a more granular analysis by examining how positional RAE patterns vary across individual leagues. Significant effects were observed among goalkeepers in the Serie A (V = 0.25), Süper Lig (V = 0.21), and LaLiga (V = 0.24) leagues; among defenders in LaLiga (V = 0.19) and Jupiler Pro League (V = 0.15); among midfielders in Serie A (V = 0.19), Ligue 1 (V = 0.14), Süper Lig (V = 0.15), and Eredivisie (V = 0.24); and among forwards in Ligue 1 (V = 0.18), Bundesliga (V = 0.18), and Scottish Premiership (V = 0.22). These findings suggest that while the RAE is present across all playing positions, its magnitude and distribution differ depending on the competitive and structural characteristics of each league.

Romann and Fuchslocher [70], observed Q2 overrepresentation among Swiss goalkeepers, while Figueiredo et al. [14], reported Q1 dominance across all positions in elite Brazilian football. In contrast, Işın and Melekoğlu [71], found no RAE among U-17 goalkeepers, possibly due to small sample sizes.

Other research has also shown that the RAE may vary by playing position, level, or region [60, 69, 72, 73]. The presence of the RAE has also been documented across various sport disciplines. Studies in handball [65, 74, 75], basketball [76], volleyball [66], rugby [77, 78], and futsal [79, 80] have all demonstrated the existence of the RAE, with variation observed by playing position within each sport.

Recent studies suggest that grouping athletes by developmental age rather than chronological age may help mitigate the effects of the RAE [81, 82]. One such strategy is birthday-banding, which allocates players to age categories based on their actual birthdates instead of rigid calendar-year cut-offs. This method, shown to reduce RAE-related disparities in talent development [68], aims to ensure fairer selection opportunities by avoiding seasonal age advantages. Another effective approach is bio-banding, which groups players according to their biological maturity rather than chronological age, helping to account for differences in physical development [83–85]. Such strategies level the playing field, particularly in early talent identification stages, and represent promising interventions to reduce or eliminate RAE-driven selection biases in sport.

### Limitations

This study has several limitations. First, it is based on secondary data, which may contain inaccuracies. Second, the cross-sectional design precludes causal interpretation. Third, the focus on male footballers in UEFA’s top ten leagues limits generalisability to lower-tier leagues, other regions, and female athletes. Lastly, the analysis does not account for cultural, socio-economic, or coaching differences between leagues, which may influence RAE patterns.

### Implications for practice and future research

Future research should adopt longitudinal designs and include more diverse samples to better understand the dynamics of the RAE. Exploring cultural, socio-economic, and coaching factors may also offer valuable insights [86, 87]. Football governing bodies, such as UEFA, could support educational programmes for coaches and scouts to raise awareness of RAE-related biases and promote potential-based selection.

While this study employed chi-square tests, odds ratios, and Cramér’s V due to the categorical structure of the data, future research may benefit from Poisson regression modelling. This method allows for the estimation of incidence rate ratios and better handling of interaction effects (e.g., league × position), as shown in recent RAE studies [34, 64, 83, 88] —which may enable more robust analyses of RAE patterns. By implementing strategies like bio-banding and birthday-banding, organisations can promote fairer talent development and broader inclusion across youth and professional football.

## Conclusion

This study analysed the presence of the RAE among professional male footballers competing in UEFA’s top ten leagues during the 2023/24 season, focusing on differences by player position and league. The findings showed a consistent overrepresentation of Q1-born players compared to Q4-born players, with the effect evident across all leagues except the Premier League. The overall effect size was small, indicating that while statistically significant, the magnitude of the RAE was limited. The RAE was also observed across all playing positions, with goalkeepers showing the most pronounced disparity. For goalkeepers, the effect size was close to moderate, whereas for other positions it remained small. League-specific positional analysis revealed significant RAE patterns among goalkeepers in Serie A, Süper Lig, and LaLiga; defenders in LaLiga and Jupiler Pro League; midfielders in Serie A, Ligue 1, Süper Lig, and the Eredivisie; and forwards in Ligue 1, Bundesliga, and Scottish Premiership. Across these contexts, the observed effect sizes ranged from small to moderate, indicating that while the RAE was present, its strength varied depending on league and playing position. These results highlight the varying magnitude of the RAE depending on positional and league contexts, underscoring the need for tailored strategies to mitigate its effects in elite football.

## Data Availability

The raw data used in this study were obtained from publicly available online sources. The dataset processed for analysis is summarized in the tables in the published paper. Readers can access the original raw data from the official online sources referred to in the methodology section, taking into account the dates of data extraction. The data that support the findings of this study, in their processed form, are available upon reasonable request from the corresponding author.

## Declarations

### Previous presentations

A preliminary version of this study was presented as an abstract at the 22nd International Sports Sciences Congress (22. Uluslararası Spor Bilimleri Kongresi), held at Gazi University, Ankara, Turkey, on November 21–24, 2024. However, the full results and analyses presented in this manuscript are original and have not been published elsewhere.

## Abbreviations

RAE: Relative age effect
UEFA: Union of European Football Associations
U-14 to U-20: Players under the ages of 14 to 20
Q1–Q4: First to fourth quarters of the year
